# Antihyperalgesic effect of joint mobilization requires Cav3.2 calcium channels

**DOI:** 10.1186/s13041-023-01049-3

**Published:** 2023-07-18

**Authors:** Daniel F. Martins, Victor Sorrentino, Leidiane Mazzardo-Martins, William R. Reed, Adair R. S. Santos, Vinícius M. Gadotti, Gerald W. Zamponi

**Affiliations:** 1 Departments of Clinical Neurosciences, and Physiology & Pharmacology, Hotchkiss Brain Institute, Alberta Children’s Hospital Research Institute, University of Calgary, Calgary, AB T2N 4N1 Canada; 2 Experimental Neuroscience Laboratory (LaNEx), Postgraduate Program in Health Sciences, University of Southern Santa Catarina, Palhoça, SC Brazil; 3 Programa de Pós-Graduação em Neurociências, Centro de Ciências Biológicas, Universidade Federal de Santa Catarina, Campus Universitário-Trindade, Florianópolis, SC Brazil; 4 Department of Physical Therapy, Rehabilitation Science Program, University of Alabama at Birmingham, Birmingham, AL USA

**Keywords:** Joint mobilization therapy, Cav3.2 channel, Mechanical hyperalgesia, Analgesia

## Abstract

The present study was undertaken to explore the relative contributions of Cav3.2 T-type channels to mediating the antihyperalgesic activity of joint manipulation (JM) therapy. We used the chronic constriction injury model (CCI) to induce peripheral neuropathy and chronic pain in male mice, followed by JM. We demonstrate that JM produces long-lasting mechanical anti-hyperalgesia that is abolished in Cav3.2 null mice. Moreover, we found that JM displays a similar analgesic profile as the fatty acid amide hydrolase inhibitor URB597, suggesting a possible converging mechanism of action involving endocannabinoids. Overall, our findings advance our understanding of the mechanisms through which JM produces analgesia.

**Supplementary Information:**

The online version contains supplementary material available at 10.1186/s13041-023-01049-3.

## Main text

Joint mobilization (JM) is a common non-allopathic treatment approach used by healthcare professionals for the management of different types of pain such as that arising from inflammation and neuropathies. Mobilization-induced analgesia has been demonstrated in human pain patients [1], and in animal models [2–4]. In recent years, evidence has emerged demonstrating that JM may exert its antihyperalgesic action via endocannabinoid signaling. Indeed, the activation of the spinal cannabinoid CB1 and peripheral CB2 receptors appears to play an important role in this antihyperalgesic action of JM [5]. Cannabinoid molecules have emerged as potential therapeutics for pain management [6]. Natural and synthetic cannabinoids, as well as endogenous cannabinoids such as anandamide and 2-AG reduce pain in animal models and humans [7–11]. Outside the brain, many of these molecules produce analgesia by inhibiting Cav3.2 T-type calcium channels expressed in the spinal cord and dorsal root ganglia, rather than just acting via cannabinoid receptors [8–10, 12–14]. Thus, we set out to test whether JM mediated analgesia may involve the Cav3.2/endocannabinoid axis.

All animal care and experimental procedures were carried out in accordance with the National Institutes of Health’s Animal Care Guidelines (NIH publications No. 80-23) and conducted following approval of animal protocols by the Ethics and Institutional Animal Care and Use committees. Ten-week-old male C57BL/6J (wild-type) and male Cav3.2 knockout mice (25–30 g) were purchased from Jackson Laboratories. Stock aliquots of drugs were dissolved in dimethylsulfoxide (DMSO) with a final DMSO concentration of no more than 5% in injection solutions. Control animals received the same vehicle used to dilute the compounds. Drugs were delivered by the intraperitoneal (i.p.) route at a volume of 10 mL/kg of body weight. We used a sciatic nerve constriction model (CCI) to induce chronic neuropathic pain. Briefly, mice were anaesthetized (isoflurane 5% induction, 2.5% maintenance) and the right sciatic nerve was exposed at the level of the thigh by blunt dissection through the biceps femoris. Proximal to the sciatic nerve trifurcation, about 10 mm of nerve was freed of adhering tissue and three ligatures (silk suture 6–0) were loosely tied around it with about 1–2 mm spacing so that the epineural circulation was preserved. In sham-operated mice, the nerve was exposed but not injured.

Mice were tested for withdrawal thresholds to mechanical stimuli using von Frey filaments (Stoelting, Chicago, USA) applied to the plantar side of the hindpaw and scored before CCI and 30 min after JM treatment on the 7th day after surgeries. Animals were kept individually in transparent plastic glass boxes set on an elevated wire grid platform that allows access to their site of testing on the hind paw. Each right hind paw was stimulated for 1 s using a 0.4 g von Frey filament. The percentage of withdrawal to 10 stimulations was considered as the nociceptive response. All experimenters were male.

**Fig. 1:**
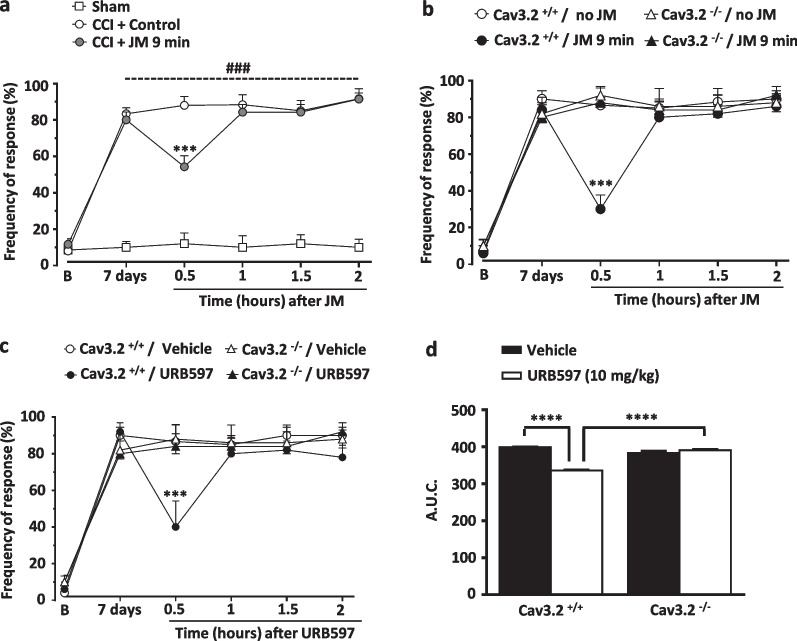
Joint mobilization reverses mechanical hypersensitivity induced by CCI. **a** Time course of the effect of a 9-min of acute ankle joint mobilization on mechanical hypersensitivity in neuropathic mice. Each point represents the mean of 5–7 animals, error bars denote S.E.M. Data are representative of 2 independent sets of experiments. Statistical analyses were performed by two-way ANOVA followed by a Tukey's test. Asterisks denote a significant difference of ***P < 0.001 when compared with the control group, and hashtags denote P < 0.001 for comparison with the sham-operated group. **b** Time-course of the effect of 9-min joint mobilization treatment in either Cav3.2 null mice or wild-type mice subjected to CCI. Each data point represents the mean of 5–6 mice and data are representative of 2 independent sets of experiments. This particular experiment was performed with the experimenter being blinded to genotype. Statistical analyses were performed by two-way ANOVA followed by a Tukey's test. Asterisks denote a significant difference of ***P < 0.001 when compared with the control group. **c** Time-course and (**d**) bar graph representing the effect of URB597 (10 mg/kg, i.p.) on nocifensive responses of neuropathic wild type and Cav3.2 null mice, expressed as area under the curve (A.U.C.). Each point or bar represents the mean of 5–6 mice, error bars are SEM. Data are representative of 2 independent sets of experiments. Statistical analyses were performed by two-way ANOVA followed by a Tukey's test. Symbols denote a significant difference of **P < 0.01, ***P < 0.001, or ****P < 0.0001 when compared with the control group

As shown in Fig. 1a, the CCI model manifested itself as long-lasting mechanical hypersensitivity to von Frey stimulation of the ipsilateral paw. We then assessed to what extent JM could alter this pain response 7 days after the establishment of neuropathy. Under light isoflurane anesthesia, the knee joint was stabilized, and the ankle joint was rhythmically flexed and extended to the end of the range of movement (animals that did not receive JM were subjected to the same isoflurane anesthesia). The treatment group received three applications of mobilization which each lasted three minutes and which were separated by 30 s of rest. As seen in Fig. 1a, such a 9-min JM treatment induced a transient relief from mechanical hypersensitivity that abated after one hour. To determine whether this effect involved Cav3.2 T-type calcium channels, we performed analogous experiments in Cav3.2 null mice which are known to have compensatory mechanisms that allow them to develop some types of chronic pain (see Fig. 1b). In this batch of mice, wild type animals exhibited robust JM-induced relief from mechanical hypersensitivity, whereas Cav3.2 null mice did not respond to JM. The involvement of Cav3.2 channels in CCI was confirmed further by systemic (intraperitoneal) delivery of 10 mg/kg of the non-selective T-type channel blocker mibefradil, whereas mibefradil failed to act in Cav3.2 null mice (Additional file 1: Fig. S1a, b). In contrast with 9-min JM treatment, a shorter duration of JM (3 min) did not mediate pain relief.

A blinded, randomized controlled trial of 31 healthy human subjects measured anandamide (AEA) levels pre- and post- manipulative treatment (MT) including JM. In subjects receiving MT, serum levels of AEA obtained after MT more than doubled while there was no change in control subjects [15]. Because Cav3.2 channels are inhibited by the endocannabinoid anandamide, it is thus possible that anandamide might participate in the actions observed in our mouse cohorts. To determine whether AEA is capable of mediating analgesia in the CCI model, we systemically delivered 10 mg/kg URB597. This compound is a selective and irreversible inhibitor of fatty acid amide hydrolase, a key enzyme responsible for the breakdown of AEA. As shown in Fig. 1c, d, URB597 mediated a transient relief from mechanical hypersensitivity in wild type mice, but not in Cav3.2 null mice. These data fit with the idea that endogenous AEA is capable of mediating pain relief via Cav3.2 channels. Given that JM was equally blunted in mice lacking Cav3.2 and the fact that endocannabinoids have been previously linked to JM [5, 15], it is thus conceivable that JM induces pain relief by triggering increased production of endocannabinoids such as AEA, which then blocks Cav3.2 channels. However, we did not directly measure AEA levels in our CCI mice, and we did not determine whether degradation of endogenous anandamide blunts the effects of JM. Hence additional work will be needed to establish a causal link between JM mediated anandamide production and an ensuing inhibition of Cav3.2 channels.

In summary, this study represents the first direct demonstration of the role of Cav 3.2 T-type calcium channels in the antihyperalgesic effect of JM.

## Supplementary Information

**Additional file 1: Figure S1 (a)** Time course of mibefradil mediated reversal of mechanical hypersensitivity in mice with a chronic constriction injury of the sciatic nerve. Statistical analyses were performed by two-way ANOVA followed by Tukey's test. Asterisks denote a significant difference of **P < 0.01 and ***P < 0.001 when compared with the control group (n = 5–7). Hashtags denote P < 0.001 for comparison with the sham-operated group. **(b)** Mibefradil at 10 mg/kg inhibits pain responses in CCI operated wild type mice, but not in Cav3.2 null mice *P < 0.05 and ***P < 0.001 (n = 5–6).

## Data Availability

All data generated or analysed during this study are included in this published article.

## Abbreviations

JM: Joint mobilization
MT: Manual therapy
CCI: Chronic constriction injury
AEA: Anandamide
DMSO: Dimethylsulfoxide
