# Supplementary figures and images for: Antihyperalgesic effect of joint mobilization requires Cav3.2 calcium channels
Source: Mol Brain. 2023 Jul 18;16:60. doi: 10.1186/s13041-023-01049-3 (PMC10355051; doi:10.1186/s13041-023-01049-3)

**a**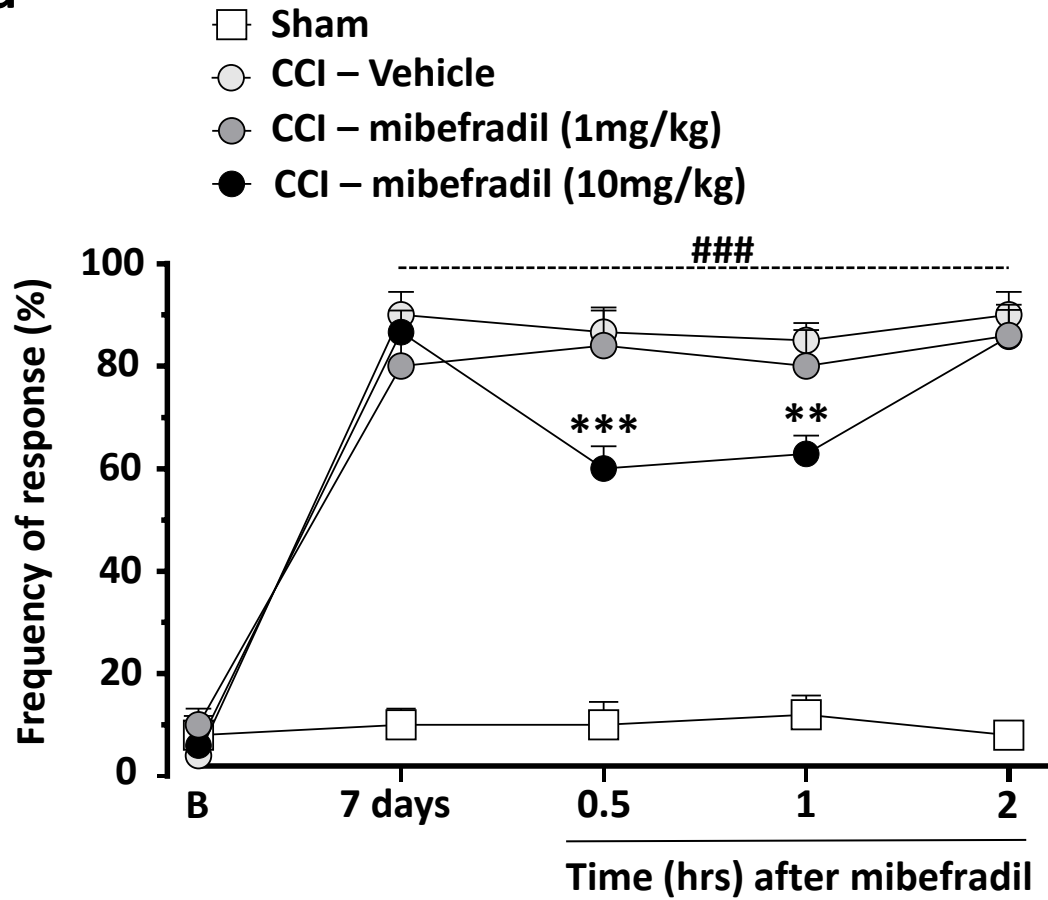**b**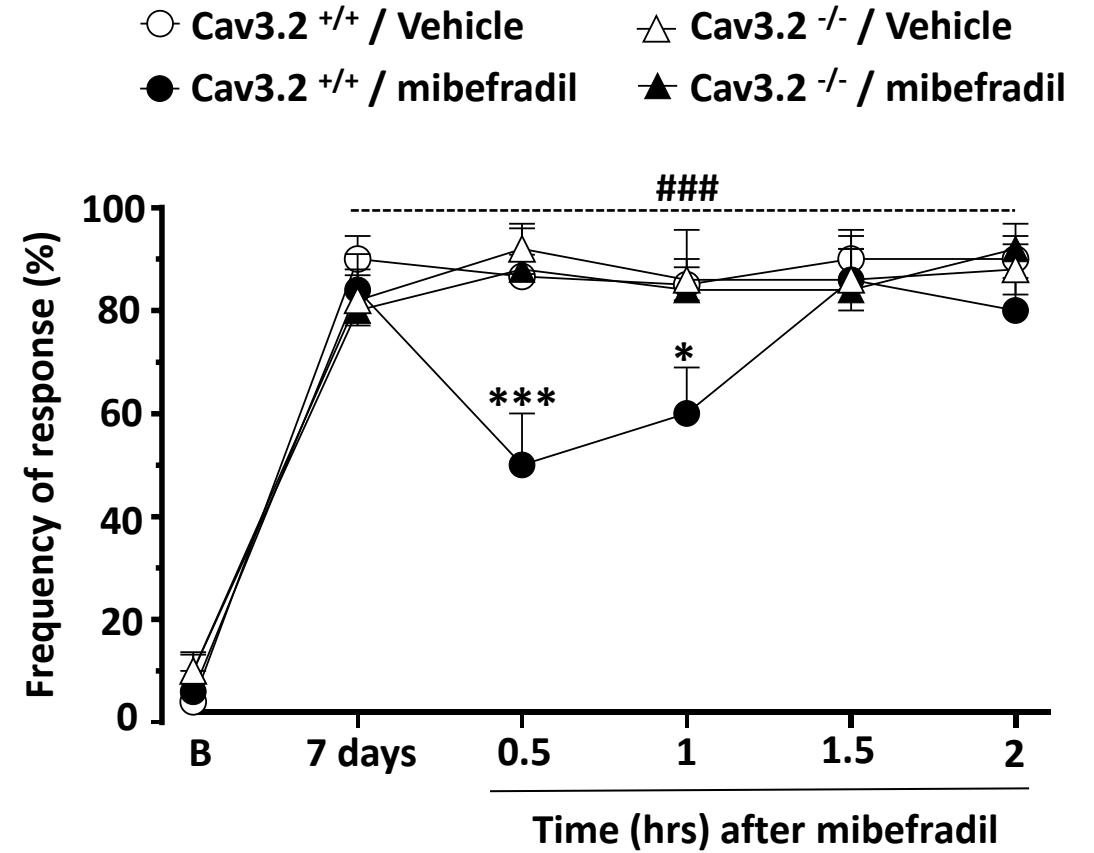

Supplement: Supplementary file 1 — Additional file 1: Figure S1 (a) Time course of mibefradil mediated reversal of mechanical hypersensitivity in mice with a chronic constriction injury of the sciatic nerve. Statistical analyses were performed by two-way ANOVA followed by Tukey's test. Asterisks denote a significant difference of **P < 0.01 and ***P < 0.001 when compared with the control group (n = 5–7). Hashtags denote P < 0.001 for comparison with the sham-operated group. (b) Mibefradil at 10 mg/kg inhibits pain responses in CCI operated wild type mice, but not in Cav3.2 null mice *P < 0.05 and ***P < 0.001 (n = 5–6). [file 13041_2023_1049_MOESM1_ESM.pdf]
